# Thermo-, Radio- and Photostability of Perindopril Tert-butyloamine in The Solid State. Comparison to Other Angiotensin Converting Enzyme Inhibitors

**Published:** 2017

**Authors:** Anna Wzgarda, Katarzyna Dettlaff, Martyna Rostalska, Ewa Pabian, Katarzyna Regulska, Beata Jadwiga Stanisz

**Affiliations:** a *Department of Pharmaceutical Chemistry, Faculty of Pharmacy, Poznan Unversity of Medical Sciences, 6 Grunwaldzka St., 60-780 Poznan, Poland. *; b *The Oncology Center of Wielkopolska, 15 Garbary St., 61-866 Poznan, Poland.*

**Keywords:** Perindopril tert-butyloamine, Stability, Photostability, Radiodegradation, ACE-I

## Abstract

The main aim of this study was determination of thermo- radio- and photostability of perindopril tert-butyloamine (PER) therefore the efficiency and safety of the therapy could be maintained.

A chromatographic method (RP-HPLC) had been validated before use to determine PER loss. The evaluation of stability properties of PER in solid state under the influence of isothermal condition, relative humidity - RH = 0% and 76.4%, exposure to 6 mln lux h and ionizing radiation generated by beam of electrons of 25–400 kGy was investigated.

Studies pointed out that presence of moisture changes a kinetic model of PER degradation; lack of moisture in the air generates a first-order kinetic model of the reaction, increase humidity generates the autocatalytic model. PER proved to be resistant for ionizing radiation. It is possible to use radiation sterilization and decontamination (dose 25 kGy) with no significant loss of content. Investigation of PER photostability proved, that after exposure to 6 mln lux h physicochemical parameters are acceptable. Among all the ACE-I, PER has one of the shortest t_0,5_. PER should be stored in closed containers, protected from high temperature and moisture. PER is referred to be photostable and resistant for radiodegradation.

## Introduction

**Figure 1:**
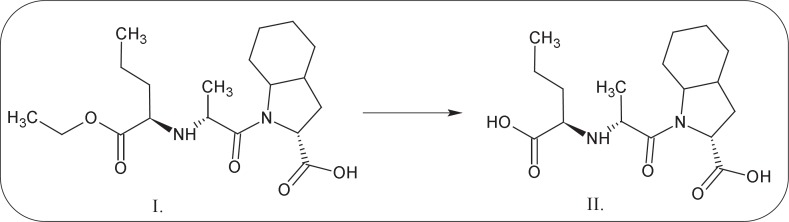
Conversion perindopril (I) to perindoprilat (II).

Perindopril (PER) belongs to the group of angiotensin converting enzyme inhibitors (ACE-I) and is widely used by patients with hypertension and heart failure. Chemical definition of PERis(2*S*,3a*S*,7a*S*)-1-[(2*S*)-2-[[(2*S*)-1-ethoxy-1-oxopentan-2-yl]amino]propanoyl]-2,3,3a,4,5,6,7,7a-octahydroindole-2-carboxylic acid (Figure1-I) and was first synthesized by Vincent *et al.* in 1982 (1). Following oral administration, it is rapidly metabolized in the liver by hydrolysis to an active metabolite-perindoprilat (Figure 1.-II). The bioavailability of pro-drug ranges between 65.6% and 95.1%; in plasma as active metabolite is present only 16.8% of an oral dose of perindopril. Perindoprilat is a competitive and potent ACE-I, the enzyme responsible for the conversion of angiotensin I to angiotensin II (2).

PER is in common use as antihypertensive agent, moreover it has been reported that long term administration of ACE-I may have more positive sides: protection against cancer (3-6), prevention of type 2 diabetes (7, 8) and delayed the onset and progression of prominent left ventricle dysfunction among children with Duchenne Muscular Dystrophy (9).

In medicine PER is used in solid state in tablet forms. Until now there were no available reports on the radiostability and photostability of PER and no existing data evaluating kinetic or thermodynamic parameters of its degradation in solid state in the worldwide literature. Stability testing is an integral part of quality control, it contributes to optimization of storage and economization of manufacture process, particularly when a pharmaceutical ingredient is unstable. The drug stability is also fundamental from clinical point of view. Any change can decline the quality of active ingredient, *ipso facto *effectiveness and safety of the therapy. Therefore, innovative results presented in the investigation have great importance for the pharmaceutical industry and patient treatment. 

Drug degradation depends on two factors: temperature level and relative air humidity (RH). First factor- temperature - induces thermal acceleration of chemical reactions. The second factor - RH can also participate in chemical process leading to hydrolysis, hydration, and other reactions, in which more RH can also contribute to the drug degradation by forming moist layer and dissolution of the active ingredient. Especially substances vulnerable to hydrolysis, as esters, should be investigated to their sensitivity to temperature and RH. The travel of every drug commences at the site of manufacture, and passes through wholesales, warehouses, drugstores and sometimes long way in different conditions before reaching the patient. That is why the control of stability of a chemical compound in different conditions is a crucial problem for pharmaceutical industry, medicines have to be kept in an environment that maintains their efficacy (10, 11). 

The radiochemical stability of PER has not been studied so far. Sterilization of drugs by using gamma or e-beam ionizing irradiation has been recommended by the European Pharmacopoeia 7th Edition and are becoming increasingly common. It is estimated that most of the substances in the solid state may be sterilized by irradiation, nevertheless, it is necessary to determine that the standard dose of 25 kGy does not damage the drug structure. The doses used in experiment (25−400 kGy) were designed to study the stability and in order to determine if it might be sterilized by irradiation (12). 

Perindopril chemically is an ester susceptible to hydrolysis, what improves its pharmacokinetic profiles and conversion to perindoprilat, however, this fact increases the liability for decomposition. PER in solid state was investigated, whereas on pharmaceutical market is available in solid state, in form of tablets. The present study aims at the stability assessment of PER in terms of influence of the air relative humidity and temperature in its decomposition process. New information about kinetic model of degradation that this study provides can induce investigations of novel pharmaceutical formulations or storage conditions (13-17).

## Experimental

*Chemicals*

Perindopril erbumine was supplied by Bachem, (serial number L-2780-004-020). Acetonitrile (Merck, Germany) and methanol (Merck, Germany) were HPLC grade. All other chemicals were of analytical reagent grade. Potassium dihydrogen phosphate (KH_2_PO_4_, M = 136,09 g/mol) and sodium chloride were obtained from POCH (Gliwice, Poland). Oxymethazoline were purchased from Sigma-Aldrich, USA. Water used throughout the study was freshly bidistilled.

*Instruments*

The chromatographic separation was achieved using LiChroCART^®^ 250-4 HPLC-Cradridge column, LiChrospher^®^ 100 RP-18 (5 µm) (Merck, Germany), which worked at ambient temperature. Entire chromatographic system consisted of a Shimadzu LC-6A Liquid Chromatograph pump with a 7725 Rheodyne value injector 20 µL fixed loop equipped with a Shimadzu SPD-6AV UV-VIS Spectrophotometric Detector. The detector was set at 215 nm and peak areas were integrated by Shimadzu C-R6A Chromatopac integrator.

*Solutions*

The applied mobile phase consisted of acetonitrile-phosphate buffer (0.001 mol/L, adjusted to pH 2 with *orto*-phosphoric acid) (70:30, v/v). It was filtered through a filter 0.22 µm and degassed by ultrasound prior to use. Oxymethazoline was dissolved in methanol at the concentration of 0.025 mg/mL served as the internal standard (I.S.) applied throughout the study. Aqueous phosphate buffer was prepared by dissolving 68.1 mg of KH_2_PO_4 _in 450 mL of bidistilled water. It was adjusted to pH 2.0 using phosphoric (V) acid (85%) and completed to 500 mL with bidistilled water.

**Figure 2:**
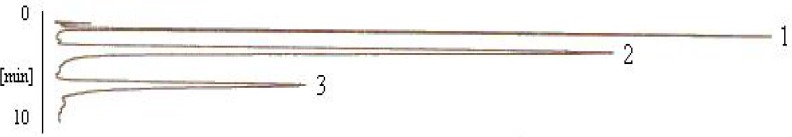
RP-HPLC chromatogram for PER (2), its degradation product (1) and internal standard (3) stored at RH = 76.4%, T = 363 K.

**Figure 3:**
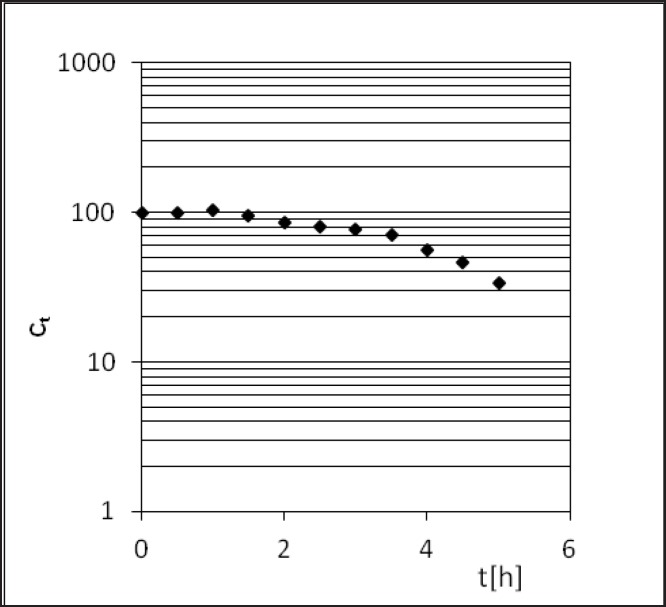
Semi-logarithmic diagram of PER decomposition during the stress test under conditions of T = 363 K and RH = 76.4%.

**Figure 4:**
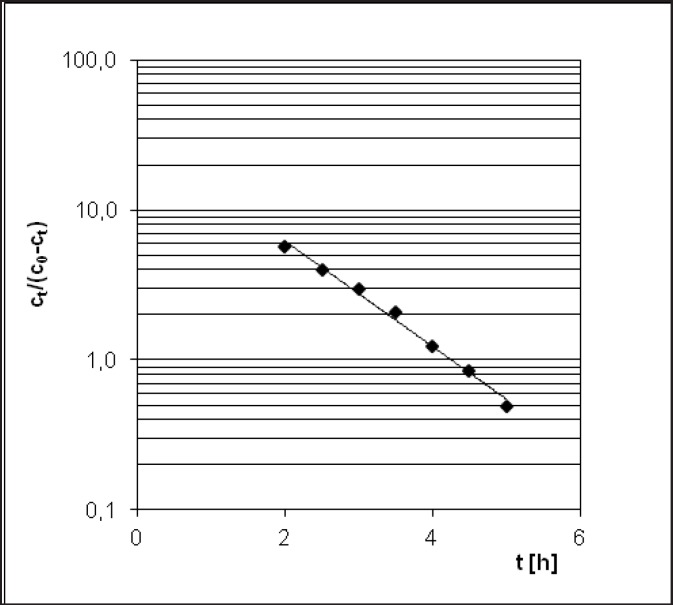
Prout-Tompkins transformation of PER decomposition diagram

**Figure 5.**
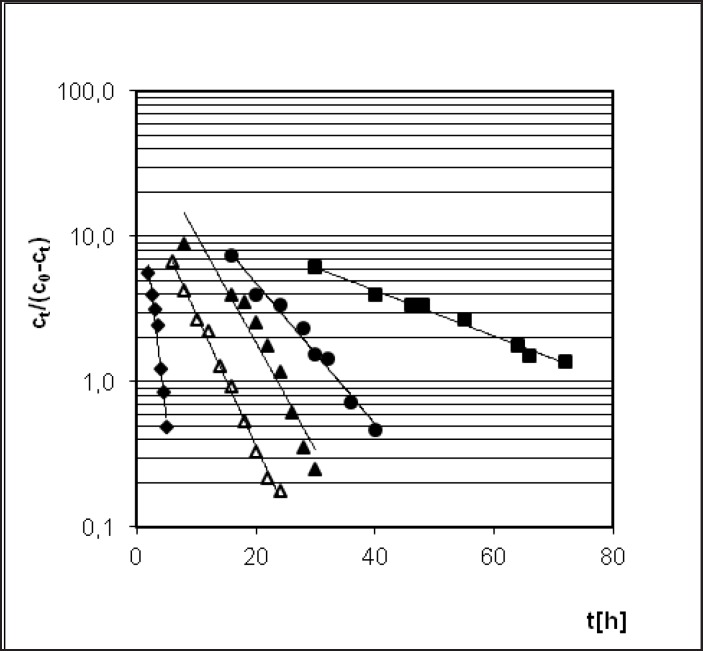
Semi-logarithmic degradation profile of PER expressed by Prout-Tompkins equation (acceleration stages of the reaction) – the influence of variable temperatures: 333 K (), 343 K (), 348 K (), 353 K (∆) and T = 363 K () in the stable value of air humidity 76.4%.

**Figure 6:**
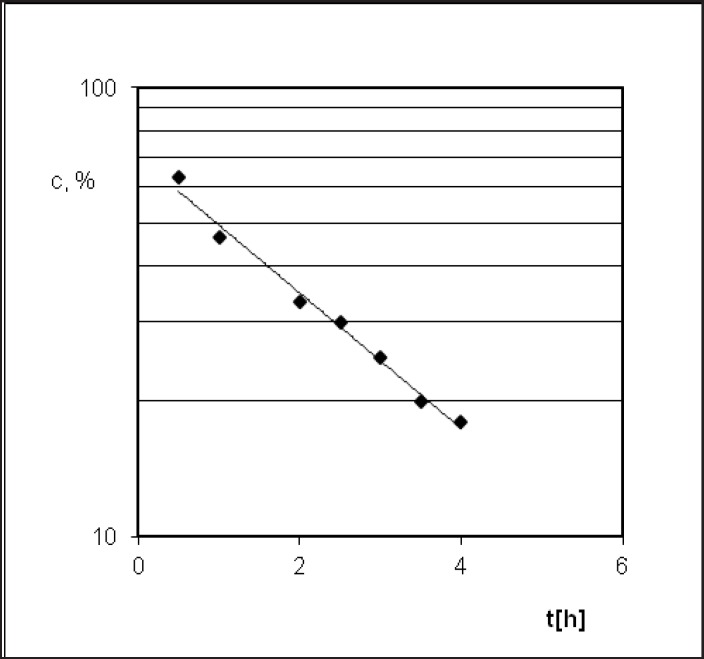
Semi-logarithmic decomposition curves in RH = 0%, where () is PER concentration during the stress test in temperature 383 K

**Figure 7:**
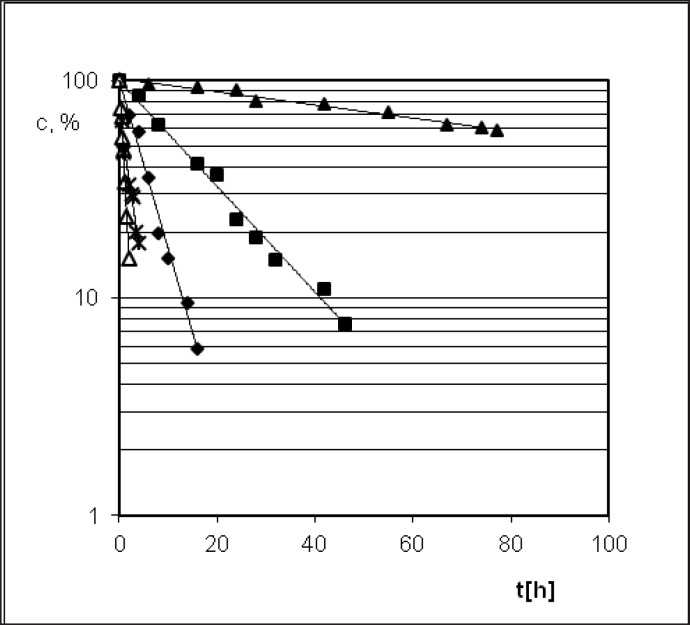
Semi-logarithmic decomposition curves in RH = 0%, where (▲) is PER concentration during the stress test in temperatures: 343 K, ()353 K, ()363 K, (x) 373 and (∆) in 383 K

**Figure 8:**
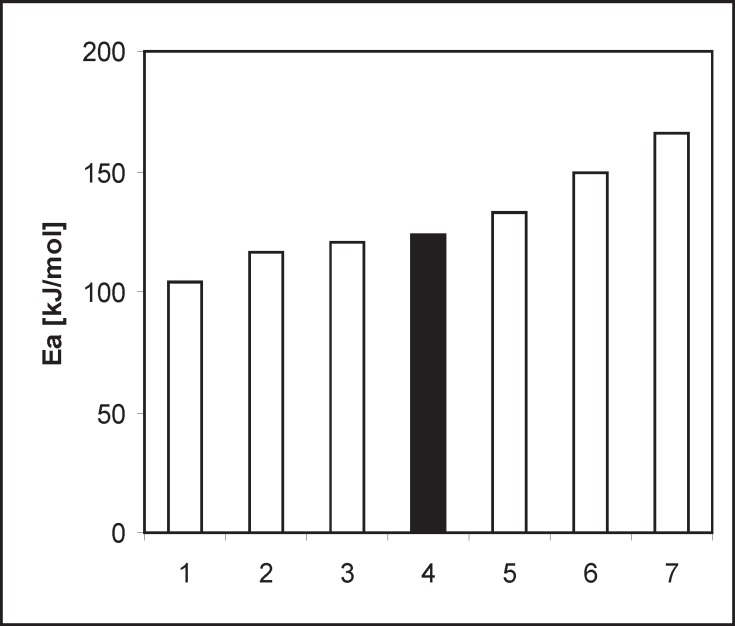
Comparison of the Ea of the decomposition reaction dicarboxylic ACE-I in the solid phase

**Table 1:** Results of validation.

**Parameters**	**Results**
Linearity range (mg/mL)	0.04 – 0.4
*Regression equation * *y = ax*	
Slope a ± Δa	2.706 ± 0.09
Standard deviation of the slope (Sa)	0.043
Standard deviation (SD)	0.015
Correlation coefficient (r)	0.998
Limits of detection (LOD)	0.018
Limit of quantification (LOQ)	0.055
Precision - low; high (%)	1.55; 2.39
Recovery (%)	99.86 ± 0.5

**Table 2:** PER degradation rate constant k [1/s] in isothermal conditions of constant air humidity R = 76.4% and different temperature values, with parameters of linear Arrhenius relationship and thermodynamic parameters: activation energy E_a_ = -aR, enthalpy ∆H^≠^ = E_a_ –RT, entropy ∆S^≠^ = R(lnA-ln[k_B_T/h].

**T**	**k ± ∆k [1/s]**	**r**	**linear Arrhenius relationship** **f(1/T) = lnk**	**Thermodynamic parameters**
333 K	(1.005 ± 0.115) 10^-5^	-0.993	*a = -*12027.594 ± 2583.648	*Ea*[kJ/mol] 124.22 ± 14.12
343 K	(3.101 ± 0.415) 10^-5^	-0.991	*Sa = *1027.97	
348 K	(4.727 ± 1.093) 10^-5^	-0.966	*b =24.588 *± 8.21	*∆H* ^≠^ * [kJ/mol*] 121.74±14.39
353K	(5.768 ± 0.313) 10^-5^	-0.997	*Sb = 2.957*	
363 K	(2.256 ± 0.359) 10^-4 ^	-0.987	*r = -0.989*	*∆S* ^≠^[J/mol*∙*K] 18.974*±*133.67

**Table 3:** PER degradation rate constant k [1/s] in isothermal conditions of constant air humidity R = 0% and different temperature values, with parameters of linear Arrhenius relationship and thermodynamic parameters: activation energy E_a_ = -aR, enthalpy ∆H^≠^ = E_a_ –RT, entropy ∆S^≠^ = R(lnA-ln[k_B_T/h

T	k ± Δk [1/s]	r	linear Arrhenius relationship f(1/T) = lnk	Thermodynamic parameters
333 K	(1.931 ± 0.217) 10^-6^	-0.991	*a *= -15363.55 ± 4835.54	*Ea[kJ/mol] * 127.73± 40.20
343 K	(1.569 ± 0.168) 10-^5 ^	-0.992	*Sa *= 1741.91	
348 K	(4.954 ± 0.555) 10-^5 ^	-0.993	*b *= 32.05 ± 13.35	*Δ* *H* *≠* * [kJ/mol] * 125.26±42.68
353K	(9.537 ± 2.028) 10-^5 ^	-0.982	*Sb *= 4.80	
363 K	(2.601 ± 0.353) 10^-4^	-0.990	*r *= -0.981	*Δ* *S* *≠* *[J/mol* *∙* *K] * 21.62±133.92

**Table 4:** Comparison of kinetic models and degradation processes dicarboxylic ACE-I (28-35).

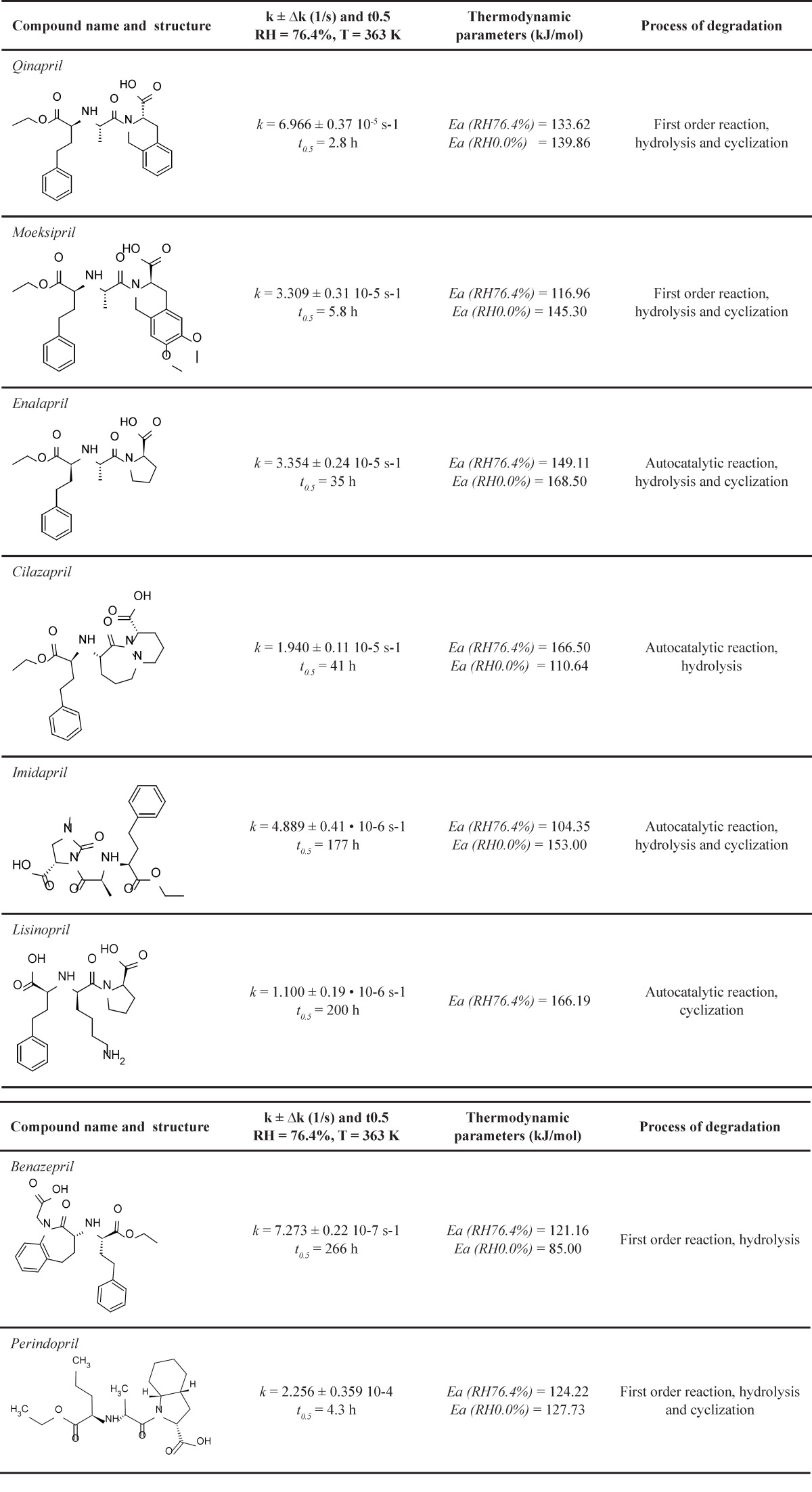

*Pretreatment of samples*

After the incubation time each sample was subjected to HPLC analysis in order to measure quantitatively the content of PER in the presence of its degradation product.

Every PER sample (10.00 mg) after the process of incubation was precisely and quantitatively transferred to a volumetric flask and subsequently filled up to 25.0 mL volume with methanol. The volume of 1.0 mL of solution obtained from each analyzed sample was mixed with 1.0 mL volume of a 0.025 mg/mL internal standard solution. The solution was subjected to HPLC.

Decrease in PER concentration in the solid state (c%) after incubation period was calculated with respect to the 0.4 mg/mL pure PER methanolic solution, considered as c = 100%, which is an equivalent of 10.00 mg pure PER sample dissolved in methanol (up to 25.0 mL) in the same way as all samples were prepared.

*Thermodegradation experiments*

A kinetic study on decomposition process of PER in solid state was carried out under isothermal conditions of increased constant relative humidity RH = 76.4% at the temperatures ranging from 333 K to 363 K and the lack of moisture RH = 0% at the temperatures 343–383 K.

Each sample consisted of 10.00 mg weighed PER in substance placed in glass, amber, uncapped vials. Prepared samples were exposed to the conditions described above. The constant, desired temperature appropriate for the test conditions (333, 343, 348, 353, 363 K for RH = 76.4% and 343, 353, 363, 373, 383 K for RH = 0%) was provided by heat chambers with the temperature control accuracy ± 1.0 K.

Appropriate relative humidity level was obtained in closed desiccators containing saturated solution of sodium chloride, which remained in contact with excess of solid salt throughout the study, obtained humidity 76.4%. The conditions of dry air (RH = 0%) were achieved by placing the sand baths in heat chamber set at the appropriate temperature. In order to equilibrate kinetic test conditions, prepared desiccators and sand baths had been put in the heat chambers at relevant temperature 24 h before the beginning of the study.

The samples were randomly removed from desiccators (RH = 76.4%) and sand baths (RH = 0%) at required time points, cooled to ambient temperature and analyzed for the extent of PER decomposition (18).

*Irradiation with e-beam radiation*

Approximately 50.00 mg of perindopril was placed in 4 mL glass jars closed with a plastic stopper and irradiated to 25, 50, 100, 200 and 400 kGy with the e-beam from a linear electron accelerator Elektronika 10/10. The energy of electrons was 9.96 MeV and the current intensity amounted 6.2 μA (19-21). 

*Calculation of radiation yield*

Radiation yield is defined as a number of molecules of reactant or newly formed products in relation to the amount of energy absorbed by the system. Radiation yield of PER degradation (G_-PER_) is defined as a ratio of decreasing number of molecules of PER as a result of irradiation with energy of 100 eV: 

$G_{-\mathrm{PER}=\frac{x}{100\mathrm{eV}}}$

G_-PER _- radiation of perindopril erbumine degradation

x - number of molecules degraded as a result of irradiation with energy of 100eV.

1 kGy = 1000 Gy 

1 Gy = 100 rad 

1 rad = 6.243 . 10^13^ eV/g

Radiation yield can be also expressed in the SI units as mol/J (19-21). 

*Photodegradation experiments*

10.00 mg of PER was placed in 4 mL glass vials and illuminated with a SUNTEST CPS+ device (Heraeus, Germany). In the photodegradation studies that were consistent with the ICH Q1B guidelines (12) the following conditions were applied: a 1500 W lamp, a 300–800 nm wavelength range, an ID65 solar filter and an irradiation intensity of 250 Wm^−2^, temperature 298 K. Exposure times of 21.6 and 108 h provided an overall illumination of not less than 1.2 million and 6 million lux h, respectively. A 10.00 mg control sample of perindopril in the glass vial was wrapped in aluminium foil.

## Results

*Validation*

The selected RPHPLC method was validated in order to confirm its applicability for this study. Precision, accuracy, selectivity, linearity, limits of quantitation and detection were evaluated according to ICH guidelines (16, 19). Results of validation are presented in Table 1.

The linearity was achieved in the concentration range between 0.4–0.04 (mg/mL). Regression equation was calculated by the least squares method and can be described by the equation: *y = ac = P*_per_*/P*_I.S._* (c [%])*, while intercept *b* from equation *y = ac + b* was statistically insignificant. The limits of detection (LOD) is described by the equation: *LOD = 3.3SD/a*, limit of quantification (LOQ) were evaluated on basis of equation: *LOQ = 10SD/a; *where *SD* stands for the standard deviation and *a* stands for the slope of the calibration curve. The method was checked for the precision and accuracy. Precision is the degree of agreement between the results obtained with the same method and on the same sample. It can be expressed as the relative standard deviations. Precision was evaluated at two levels: high and low, relative standard deviations (RSD): 

for high RSD = 2.39% and low RSD = 1.55%. Recovery values of PER from model mixtures were also adequate and amounted to 99.86 ± 0.5%.

The selectivity of the HPLC method was adequate and it chromatogram (presented on Figure 2) demonstrates separation of PER (2), degradation product (1) and internal standard (3) achieved during the chromatographic process.

The degradation product (1), which is a result of stress studies did not interfere with the detection of PER (2). The corresponding retention times were found to be about 3.5 min for product of PER degradation; 5.5 min for PER and 9.5 min for internal standard (3).

*Effect of temperature and relative humidity*

*Relative humidity = 76.4%*

PER decomposition reaction at elevated relative humidity (RH = 76.4%) and the temperature (333-363 K) takes place according to the model autocatalytic reaction (Figure 3). To describe the autocatalytic reaction mathematically, the Prout-Tompkins equation was applied. According to the concept of the Prout-Tompkins equation the acceleratory period of autocatalytic reaction after transformation gives a linear relationship (Figure 4). This can be defined by the equation: 

ln ct/(c0 – ct) = C – k * t

where *ct* stands for a concentration of PER after t [h] of incubation period, *c*_0_ stands for 100% PER concentration in solid state before the incubation, C stands for the parameter related to induction period and k stands for reaction rate constant at specified temperature and 

RH = 76.4% [1/s].

The Arrhenius equation *ln k*_i_* = lnA – E*_a_*/RT *presents the dependence between temperature T of the reaction rate constant k, where A means frequency coefficient, E_a _- activation energy and R stands for universal gas constant. In order to calculate linear Arrhenius relationship parameters the reaction rate constants for degradation of PER were determined. Samples with drug were placed under conditions of constant RH = 76.4% and T = 333, 343, 348, 353 and 363 K (Figure 5). The parameters of linear plot of Arrhenius equation (r = 0.989) enabled the evaluation of thermodynamic parameters such as activation energy, enthalpy (H), and entropy (S) (Table 2).

*Relative humidity = 0%*

PER in solid state in conditions of increased temperature (343–383 K) and the lack of moisture (RH = 0%) decomposes in a different manner than in the presence of moisture (RH = 76.4%). The curve of PER degradation was not sigmoidal, but biexponential (Figures 6 and 7).As it results from analysis of experimental data in condition of RH 0% PER is decomposed according to the first-order kinetic model 

(Table 3).

*Photodegradation test*

Exposure to light for 106 h in the Suntest CPS+ chamber, simulating the energetic composition of sunlight, was equivalent to a dose of 6 mln lux h, which was 5 times greater than the minimum value recommended for photostability studies. According to the ICH guidelines if a drug manifests its degradation after reception of a 1.2 lux h dose, it is considered as photolabile. When its content and physicochemical parameters are acceptable following exposure to 6 mln lux h, the substance is referred as photostable (13, 19). The loss of the content after the exposure to light was only 1.07% relative to the control sample, thus it may be concluded the compound turned out to be photostable. 

*E-beam radiation*

The standard dose of radiation sterilization (25 kGy) (21) did not have a destructive effect on PER (content of the PER decreased about 0.36%). It can be sterilized by e-beam radiation. Only the highest dose of radiation (400 kGy) damaged a molecule of the compound with a loss of content 9.92%. On the basis of these values radiation yield of radiodegradation of PER was calculated and found to be 5.14 molecules per 100 eV for 400 kGy (5.6 × 10^7 ^mol/J). According to the literature (21-26) radiation yield of degradation process of many drugs in the solid state is in the range of 10^6^10^8 ^mol/J, depending on the radiation dose. 

## Discussion

Knowledge about degradation conditions of the drug allows to take necessary steps to enhance stability and optimization of the pharmacotherapy in all climate zones (11, 12 and 18). Among all of the angiotensin-converting enzyme inhibitors, there is not a large number of references mentioning PER analysis in pharmaceutical dosage forms. High-performance liquid chromatography has been the major technique used for the simultaneous determination of PER and its degradation products, recommended by European Pharmacopoeia (26) and on known published HPLC methods of PER and other ACE inhibitors, may be considered to be more specific than other methods (16, 17 and 28-39), the procedure for the evaluation of PER stability in this study was properly validated as required under ICH guidelines Q2 (R1) (40). Published works about validation of the HPLC method for the determination of perindopril erbumine were focusing on PER in mixture with amlodipine (17, 38), indapamide (37), or in human plasma (39). Among other methods described for the PER determination we can find spectrophotometric method using 1-fluoro-2,4-dinitrobenzene (41) and focused more on the capillary electrophoretic separation method (42, 43).

*Stability studies of PER in solid phase*

Perindopril *tert*-butylamine has two main degradation pathways, *i.e.* the degradation by hydrolysis and the degradation by cyclization, what is presented in its monograph, in the European Pharmacopoeia, Fifth Edition (27). PER thermodegradation studies were presented as decreasing concentration in solid state sample (c[%]) plotted against the time (t[h]) (Figures 3 and 6). Analysis of those decay curves led us to the conclusion that presence of moisture in the surrounding environment influences the kinetic model of PER degradation. For the interpretation of experimental data two mathematical models were applied in order to achieve a linear model, through which, calculation of the kinetic and thermodynamic parameters of PER degradation in solid state were possible. The degradation of PER at observed conditions was shown to follow the first order kinetics in RH = 0%, and autocatalytic in RH = 76.4%. Before kinetics PER was described but in the aqueous solutions (44, 45).

*Stability of PER against the other ACE-I*

A similarity in chemistry and pharmacology of all the angiotensin converting enzyme inhibitors can be found (35). Nevertheless, there are two kinetic models of decomposition and two possible processes of degradation after exposure to increased RH: hydrolysis and/or intramolecular cyclization (46). In order to determine the location of PER against the stability of the other dicarboxylic derivatives of ACE-I, literature data were collected and presented in Table 3 and Figure 8 (28-35). Lack of intramolecular cyclization can be explained by the spatial structure of these compounds (Table 4), cyclic or bicyclic substituents close inside amide carbonyl group and a carboxyl group, and these groups participate in the cyclization reaction. Comparison of *Ea *of the decomposition reaction for ACE-I is shown in Figure 8.

## Conclusions

Evaluation through the process of method validation proved that the proposed method is suitable for the simultaneous determination of perindopril *tert*-butylamine as well as its impurities. The results of the PER stability study broaden the knowledge about process of PER degradation. It could be concluded that PER underwent a deestrification reaction forming perindoprilat in the presence of moisture, and cyclization after exposure to dry air. This fact has a negative impact on the pharmaceutical availability, because perindoprilat and cyclized form cannot be absorbed from gastrointestinal tract. Taking great instability on exposure to air humidity and high temperature into consideration, we may conclude that these factors have to be avoided during the storage process. Among all the ACE-I, PER has one of the shortest t_0,5_.

PER is resistant for ionizing radiation. That makes it possible to use irradiation sterilization and decontamination (as a dose of 25 kGy caused only a 0.36% loss of content, which probably stands for the dehydrolyzed form). Its content was observed under the increasing level of ionizing radiation and reached the highest point (9.92%) at 400 kGy.
